# PLAP expression is linked to invasive tumor growth in urothelial carcinoma of the bladder

**DOI:** 10.1007/s11255-024-04319-8

**Published:** 2024-12-16

**Authors:** Henning Plage, Kira Furlano, Sebastian Hofbauer, Florian Roßner, Simon Schallenberg, Sefer Elezkurtaj, Maximilian Lennartz, Andreas Marx, Henrik Samtleben, Margit Fisch, Michael Rink, Marcin Slojewski, Krystian Kaczmarek, Thorsten Ecke, Tobias Klatte, Stefan Koch, Nico Adamini, Sarah Minner, Ronald Simon, Guido Sauter, Joachim Weischenfeldt, Thorsten Schlomm, David Horst, Henrik Zecha, Martina Kluth, Sarah Weinberger

**Affiliations:** 1 Department of Urology, Charité – Universitätsmedizin Berlin, Corporate Member of Freie Universität Berlin, Humboldt-Universität Zu Berlin and Berlin Institute of Health, Charitéplatz 1, 10117 Berlin, Germany; 2 Institute of Pathology, Charité – Universitätsmedizin Berlin, Corporate Member of Freie Universität Berlin, Humboldt-Universität Zu Berlin and, Berlin Institute of Health, Berlin, Germany; 3 Institute of Pathology, University Medical Center Hamburg-Eppendorf, Hamburg, Germany; 4 Department of Pathology, Academic Hospital Fuerth, Fuerth, Germany; 5 Department of Urology, University Medical Center Hamburg-Eppendorf, Hamburg, Germany; 6 Department of Urology, Marienhospital Hamburg, Hamburg, Germany; 7 Department of Urology and Urological Oncology, Pomeranian Medical University, Szczecin, Poland; 8 Department of Urology, Helios Hospital Bad Saarow, Bad Saarow, Germany; 9 Department of Pathology, Helios Hospital Bad Saarow, Bad Saarow, Germany; 10 Department of Urology, Albertinen Hospital, Hamburg, Germany; 11 Biotech Research & Innovation Center (BRIC), University of Copenhagen, Copenhagen, Denmark; 12 Finsen Laboratory, Rigshospitalet, Copenhagen, Denmark

**Keywords:** Bladder Cancer, Tissue Micro Array, PLAP, Prognosis, Biomarker

## Abstract

**Purpose:**

Placental alkaline phosphatase (PLAP) is a protein with a poorly understood function that is normally only expressed in the placenta. In cancer, PLAP expression is a hallmark of germ cell neoplasms, but it can also occur in urothelial carcinoma. To evaluate the potential clinical significance of PLAP expression in bladder cancer,

**Methods:**

PLAP protein was analyzed by immunohistochemistry in more than 2500 urothelial bladder carcinomas in a tissue microarray format.

**Results:**

PLAP staining was absent in normal urothelial cells but was observed in 15.9% of urothelial carcinomas, including 282 (11.5%) with weak, 57 (2.3%) with moderate, and 51 (2.1%) with strong staining. PLAP positivity occurred in 4.1% of 413 pTa G2 low-grade, 10.2% of 176 pTa G2 high-grade, and 7.2% of 97 pTa G3 tumors (p = 0.0636). As compared to pTa tumors, the PLAP positivity rate was markedly higher in 1341 pT2-4 carcinomas (19.8%, p < 0.0001). Within pT2-4 carcinomas, PLAP staining was unrelated to pT, pN, grade, L-status, V-status, overall survival, recurrence-free survival, and cancer-specific survival (p > 0.25). However, PLAP positivity was linked to p16 positivity (p = 0.0185), GATA3 positivity (p < 0.0001), and p63 expression loss (p = 0.0456).

**Conclusion:**

In summary, these data show that PLAP is expressed in a significant fraction of pT2-4 urothelial carcinomas, unrelated to cancer aggressiveness but associated with specific molecular features. Once anti-PLAP cancer drugs become effective, urothelial carcinoma is a candidate tumor entity for clinical evaluation.

## Introduction

Bladder cancer is among the top ten most frequently diagnosed cancers globally. With around 570,000 new cases annually, it is the tenth most common cancer worldwide [1]. The majority of the patients initially presents with early-stage non-invasive (pTa) or minimally invasive (pT1) cancers, which can be treated with bladder preserving treatments, but are known for their high rates of recurrence and progression [2]. Conversely, muscle-invasive tumors (> pT2) are aggressive malignancies requiring intensive, multi-modal treatment approaches, including systemic therapies, surgery, or radiotherapy. Despite these efforts, outcomes remain variable, with nearly 50% of patients developing early metastasis and succumbing to the disease [3]. A deeper understanding of the molecular features driving disease progression could enhance the prediction of individual patient prognosis, thereby improving treatment decisions and enabling more aggressive interventions for high-risk patients.

Placental alkaline phosphatase (PLAP), also known as alkaline phosphatase, placental type (ALPP) is a 65 kDa protein coded by the ALPP gene at 2q37.1 [4, 5]. PLAP is thought to play a role in guiding migratory cells and transport specific molecules over the plasma membrane [6]. In normal tissues, PLAP expression is almost exclusively seen in the placenta where it is detectable from the 7th week of gestation and its concentration increases throughout the pregnancy [7]. Among tumors, PLAP expression is a hallmark of testicular germ cell tumors. PLAP immunohistochemistry (IHC) is thus used for the distinction of germ cell tumors from other tumor entities (summarized in [8]). Because of its membranous location, PLAP also represents a promising therapeutic target [9]. In a recent study on 12,381 tumors from 131 different tumor entities we have recently identified PLAP expression, even at high levels, in several non-testicular tumor entities for which PLAP was not expected to play a role. This included PLAP positivity in about 20% of muscle-invasive urothelial carcinomas [10].

As the clinical role of PLAP expression in urothelial cancer is unknown, more than 2,700 urothelial carcinomas were analyzed for PLAP in a tissue microarray (TMA) format in this study to search for a potential link between PLAP expression and tumor progression or patient outcome.

## Materials and methods

### Tissue microarrays (TMA)

The TMAs used in this study were first employed in a recent study on the prognostic role of GATA3 expression in urothelial bladder cancer [11]. This method enables high-throughput analysis of molecular markers across various samples. Our set of TMAs contained one sample each from 2710 urothelial tumors of the bladder archived at the Institute of Pathology, University Hospital Hamburg, Institute of Pathology, Charité Berlin, Department of Pathology, Academic Hospital Fuerth, or Department of Pathology, Helios Hospital Bad Saarow, and/or treated at Department of Urology, University Hospital Hamburg, Department of Urology, Charité Berlin, Department of Urology, Helios Hospital Bad Saarow, Department of Urology, Albertinen Hospital Hamburg (all in Germany), and Department of Urology and Urological Oncology, Pomeranian Medical University, Szczecin, Poland between 2003 and 2021. Patients at each center received treatment in line with the prevailing guidelines of the time. Specifically, those with pTa/pT1 disease underwent a endoscopic transurethral of the bladder tumo and patients with pT2-pT4 disease were treated through a radical cystectomy. Available histopathological data including grade, tumor stage (pT), lymph node status (pN), and status of venous (V) and lymphatic (L) invasion are shown in Table 1. Clinical follow up data (overall survival, cancer-specific survival, recurrence-free survival) were available from 676 patients with pT2-4 carcinomas treated by cystectomy (median: 15 months; range: 1–176 months). Immunostaining data on p53, CK20, p16, GATA3, and p63 were available from previous studies [11–13] (p63 in bladder cancer—MS submitted). The tissues were fixed in 4% buffered formalin and then embedded in paraffin. The TMA manufacturing process has previously been described in detail [14, 15]. Per patient, one cancer tissue spot (diameter: 0.6 mm) was used. The use of archived remnants of diagnostic tissues for TMA manufacturing, their analysis for research purposes, and patient data were according to local laws (HmbKHG, §12) and analysis had been approved by the local ethics committee (Ethics commission Hamburg, WF-049/09). All work has been carried out in compliance with the Helsinki Declaration.

**Table 1:** Patient cohort

	Study cohort on TMA (n = 2710)
Follow up	636
Months	
Mean	26.7
Median	15
Tumor stage	
pTa	887 (39.2%)
pT2	462 (20.4%)
pT3	615 (27.2%)
pT4	298 (13.2%)
Tumor grade	
G2	820 (30.6%)
G3	1858 (69.4%)
Lymphnode metastasis	
pN0	734 (62.0%)
pN +	449 (38.0%)
Resection margin	
R0	595 (80.6%)
R1	143 (19.4%)
Lymphatic invasion	
L0	275 (49.5%)
L1	281 (50.5%)
Venous invasion	
V0	450 (74.4%)
V1	155 (25.6%)

Percent in the column “study cohort on TMA” refers to the fraction of samples across each category. Numbers do not always add up to 2710 in the different categories because of cases with missing data

### Immunohistochemistry

For this study we used identical methods for immunohistochemical evaluation of PLAP as previously described [10]. Freshly prepared TMA sections underwent immunostaining in a single experiment conducted on the same day. Slides were deparaffinized with xylol, rehydrated through a graded alcohol series and exposed to heat-induced antigen retrieval for 5 min in an autoclave at 121 °C in Dako Target Retrieval Solution, pH9 (Agilent Technologies, Santa Clara, CA, USA; #S2367). Endogenous peroxidase activity was blocked with Dako REAL Peroxidase-Blocking Solution (Agilent Technologies, Santa Clara, CA, USA; #S2023) for 10 min. Primary antibody specific for PLAP (rabbit recombinant monoclonal, MSVA-350R, MS Validated Antibodies, Hamburg, Germany) was diluted 1:150 and applied for 60 min at 37 °C. Bound antibody was then visualized using the Dako REAL EnVision Detection System Peroxidase/DAB + , Rabbit/Mouse kit (Agilent Technologies, Santa Clara, CA, USA; #K5007) according to the manufacturer’s directions. The sections were counterstained with hemalaun. For tumor tissues, the percentage of positive neoplastic cells was estimated, and the staining intensity was semi-quantitatively recorded (0, 1 + , 2 + , 3 +). For statistical analyses, the staining results were categorized into four groups based on percentage and intensity as previously discribed [16]. Tumors without any staining were considered negative. Tumors with 1 + staining intensity in ≤ 70% of tumor cells and 2 + intensity in ≤ 30% of tumor cells were considered weakly positive. Tumors with 1 + staining intensity in > 70% of tumor cells, 2 + intensity in 31–70%, or 3 + intensity in ≤ 30% of tumor cells were considered moderately positive. Tumors with 2 + intensity in > 70% or 3 + intensity in > 30% of tumor cells were considered strongly positive.

### Statistics

Statistical analyses were conducted using JMP17^®^ software (SAS^®^, Cary, NC, USA). To identify associations between PLAP immunostaining, other molecular parameters, and tumor phenotype, we utilized contingency tables and the chi^2^-test. Kaplan–Meier curves were generated for survival analysis, and the Log-Rank test was employed to determine significant differences between groups. A p-value of ≤ 0.05 was deemed statistically significant.

## Results

### Technical issues

Of our 2710 urothelial carcinomas, 2442 (90.1%) were interpretable for PLAP. Non-interpretable tumors were caused by a lack of unequivocal tumor cells on the TMA spots or absence of entire tissue spots on the TMA.

### PLAP in urothelial carcinomas

**Fig. 1:**
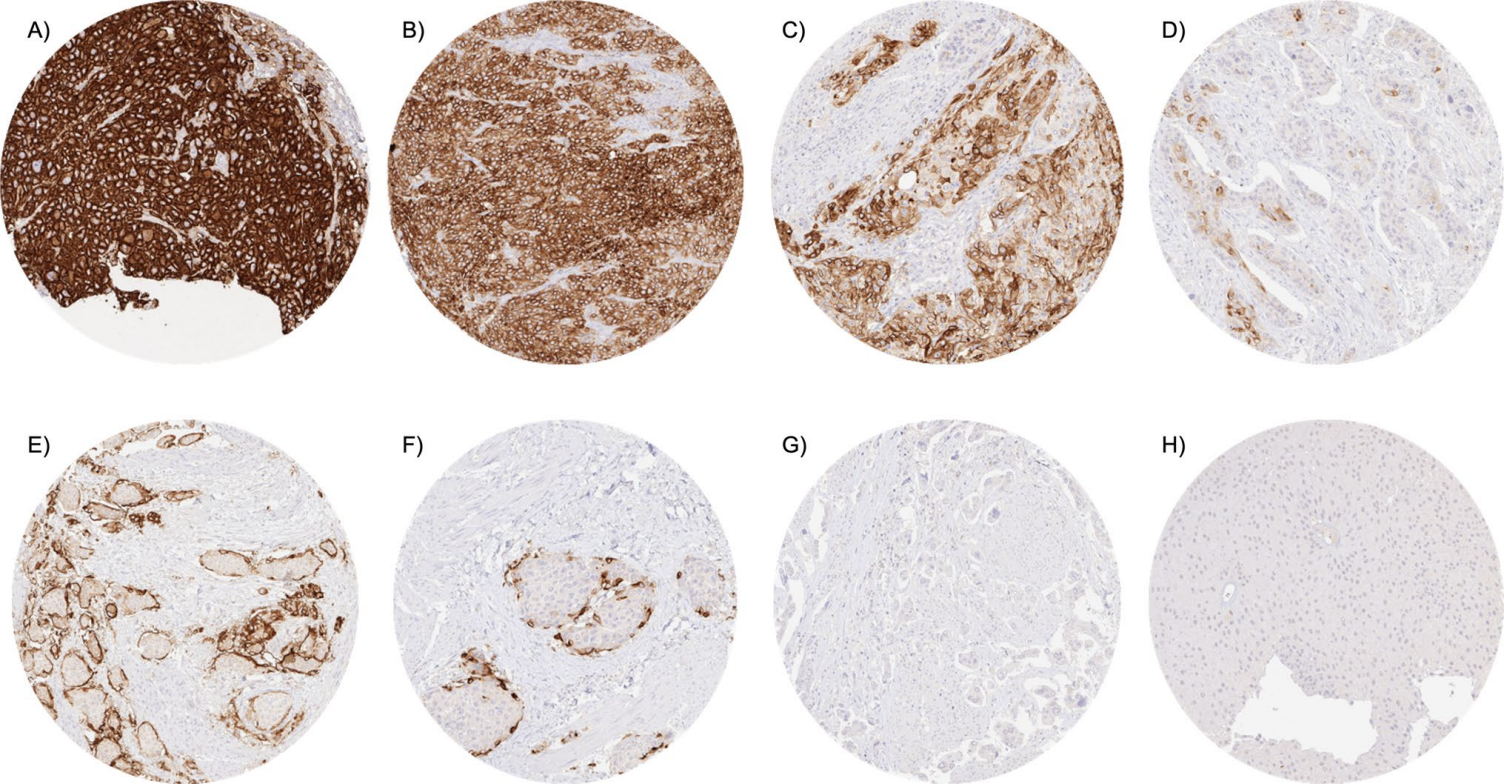
Pattern of PLAP immunostaining in urothelial carcinomas. The panels show a strong predominantly membranous PLAP staining in most cells of two muscle-invasive urothelial carcinomas (**A**, **B**), a membranous and cytoplasmic PLAP positivity of variable intensity in most cells of a pT2-4 carcinoma (**C**), a weak to moderate predominantly cytoplasmic PLAP positivity of a small subset of tumor cells in a pT2-4 urothelial carcinoma (**D**), and a strong, predominantly membranous PLAP positivity which is largely restricted to the stroma adjacent tumor cell layer in two other pT2-4 carcinomas (**E**, **F**). PLAP immunostaining is absent in a pT2-4 carcinoma (**G**) and in a pTaG2 low grade tumor (**H**)

**Fig. 2:**
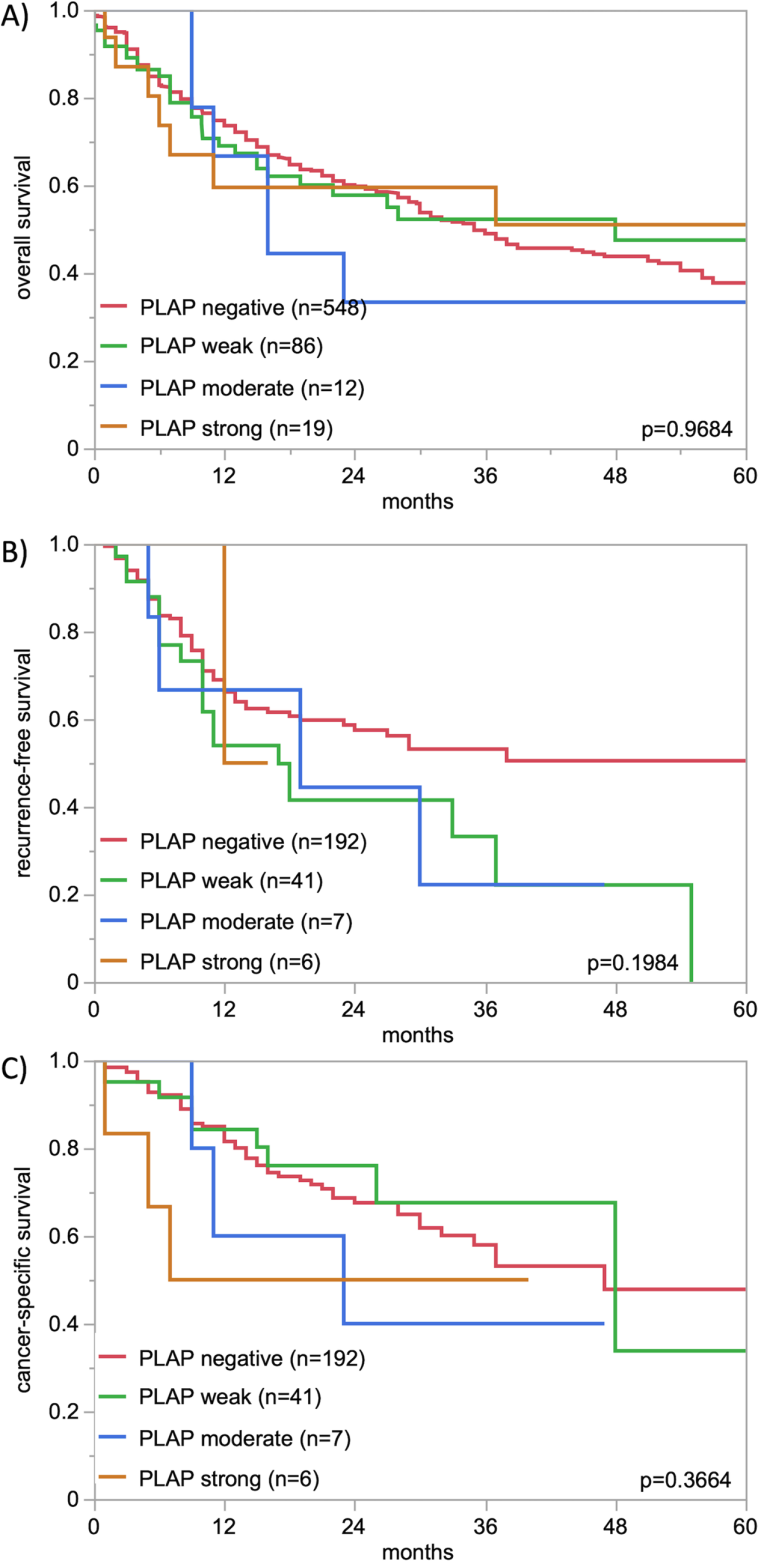
PLAP immunostaining and prognosis in muscle-invasive urothelial carcinomas. **A** Overall survival, **B** recurrence-free survival, and **C** cancer-specific survival

PLAP staining was always absent in normal urothelial cells. A PLAP positivity was observed in 15.9% of urothelial carcinomas including 282 (11.5%) with weak, 57 (2.3%) with moderate, and 51 (2.1%) with strong staining. PLAP staining was usually membranous and cytoplasmic in tumors with strong positivity while the fraction of cells with predominantly cytoplasmic staining increased in cases with low grade positivity. In a fraction of invasively growing tumors, PLAP positive cells predominated in the peripheral cell layers of invasive tumor nests. Representative images of PLAP immunostainings are shown in Fig. 1. The relationship between PLAP staining and tumor phenotype is shown in Table 2. Within pTa tumors, PLAP positivity was generally rare (6.1%) and increased only slightly from pTaG2 low-grade (4.1% positive) to pTa G2 high-grade (10.2% positive) and pTa G3 (7.2%; p = 0.0636). As compared to pTa, the PLAP positivity rate was significantly higher in muscle-invasive cancers (19.8%, p < 0.0001). However, within 1341 pT2-4 carcinomas, PLAP immunostaining was not significantly related to pT, pN, grade, L-, and V-status. Moreover, in 665 pT2-4 carcinomas that were treated by cystectomy, the PLAP staining intensity was not associated with overall, cancer-specific or recurrence-free survival (Fig. 2A–C) This also held true if subsets of pT2, pT3, and pT4 cancers were separately analyzed (data not shown).

**Table 2:** PLAP immunostaining and cancer phenotype

	n	PLAP immunostaining result				p-value
		Negative (%)	Weak (%)	Moderate (%)	Strong (%)	
All cancers	2442	84.0	11.5	2.3	2.1	
pTa G2 low	413	95.9	3.1	1.0	0.0	0.0636
pTa G2 high	176	89.8	8.5	1.1	0.6	
pTa G3	97	92.8	4.1	2.1	1.0	
pT2	445	81.6	12.8	2.5	3.1	0.6722
pT3	602	78.9	15.8	2.7	2.7	
pT4	294	81.6	11.9	3.4	3.1	
G2	105	84.8	12.4	0.0	2.9	0.0823
G3	1208	79.9	14.2	3.1	2.8	
pN0	667	82.0	12.7	1.9	3.3	0.2267
pN +	444	77.9	16.9	2.5	2.7	
R0	560	79.5	14.8	2.5	3.2	0.1154
R1	140	77.1	17.1	5.0	0.7	
L0	265	79.2	16.2	2.6	1.9	0.9180
L1	276	77.9	16.3	3.6	2.2	
V0	432	79.9	14.6	3.0	2.5	0.5519
V1	153	74.5	18.3	3.3	3.9	

*pT* pathological tumor stage, *G* Grade, *pN* pathological lymph node status, *R* resection margin status, *L*: lymphatic invasion, *V* venous invasion

*Only in pT2-4 urothelial carcinoma

### PLAP expression and molecular features in bladder cancer

In muscle-invasive urothelial carcinomas, PLAP positivity was significantly linked to p16 positivity (p = 0.0185), GATA3 positivity (p < 0.0001), and p63 negativity (p = 0.0456). No association was found with CK20 and p53 immunostaining results (Table 3).

**Table 3:** PLAP immunostaining and other molecular features

	n	PLAP immunostaining result				p-value
		Negative (%)	Weak (%)	Moderate (%)	Strong (%)	
CK20 negative	742	82.7	12.1	2.2	3.0	0.2526
CK20 weak	84	72.6	19.0	6.0	2.4	
CK20 moderate	66	75.8	18.2	4.5	1.5	
CK20 strong	384	78.4	15.9	3.1	2.6	
GATA3 negative	484	86.6	9.9	1.7	1.9	< 0.0001
GATA3 weak	304	73.0	16.8	4.9	5.3	
GATA3 moderate	241	76.8	18.3	3.3	1.7	
GATA3 strong	176	77.3	18.2	1.1	3.4	
p16 negative	562	83.1	11.0	3.2	2.7	0.0185
p16 weak	139	81.3	17.3	0.7	0.7	
p16 moderate	80	77.5	16.3	5.0	1.3	
p16 strong	465	76.1	17.2	2.8	3.9	
p63 negative	204	75.5	16.2	3.4	4.9	0.0456
p63 weak	91	75.8	15.4	2.2	6.6	
p63 moderate	164	76.8	18.9	1.2	3.0	
p63 strong	763	82.0	13.1	3.1	1.7	
p53 negative	148	79.7	12.8	2.0	5.4	0.6840
p53 very low	199	80.4	15.6	2.5	1.5	
p53 low	505	79.0	16.0	2.8	2.2	
p53 high	74	82.4	10.8	2.7	4.1	
p53 very high	277	78.7	14.1	4.0	3.2	

## Discussion

The results of this study demonstrate that PLAP expression occurs in about 15% of muscle-invasive urothelial carcinomas and that PLAP immunostaining is unrelated to clinically aggressive cancer but to specific molecular features.

The 20% positivity found in our pT2-4 carcinomas is very similar to the 22% that we previously observed in an independent analysis of a partially overlapping set of 1207 pT2-4 carcinomas [10]. The high intra-laboratory consistency of data is due to consistent staining interpretation and a highly validated staining approach. Our immunohistochemical PLAP assay has previously been validated according to the guidelines of the international working group for antibody validation (IWGAV) [17], by comparison with a second independent antibody and with RNA expression data obtained from three different publicly accessible databases in 76 different normal tissue categories [10]. The markedly higher rate of PLAP positivity and strong positivity in muscle-invasive (pT2-4) carcinomas as compared to pTa tumors in combination with the complete absence of PLAP staining in normal urothelium suggests that PLAP neo-expression occurs in a fraction of cases during development and progression of urothelial neoplasms. The lack of associations with any clinico-pathological parameters for tumor aggressiveness in muscle-invasive urothelial carcinomas does not provide evidence for a direct role of PLAP for conferring increased aggressiveness to tumor cells, however. The perceived function of PLAP as a regulator of intramembranous transport is also not indicative of an important role of PLAP in cancer.

However, other molecular features such as p53 alterations [18], HER2 overexpression [19], and MYC alteration [20] have also failed to be prognostic in most bladder cancer studies although they are known cancer-drivers and related to patient outcome in many other tumor entities [21, 22]. Parameters for luminal tumor phenotype—which was linked to favorable disease outcome in studies analyzing RNA—such as uroplakin 1a, uroplakin 1b, or GATA3 were also unrelated to patient outcome in our cohort of more than 600 muscle-invasive carcinomas in recent studies [11, 23]. It is of note, that prognosis assessment in muscle-invasive urothelial carcinomas is notoriously difficult. This tumor entity is one of only few for which not even a prognostically relevant histologic grading exists [24]. The International Society of Urological Pathology (ISUP) has therefore recommended to not grade urothelial carcinomas once they invade the muscular bladder layer [25]. Lack of a prognostic relevance of a molecular parameter is thus not indicative for absence of an important tumor biological role in urothelial cancer. The fact that de novo expression of PLAP was linked to features of aggressive disease in colorectal cancer in our previous IHC analysis of 652 cancers [10] and that RNA data from database suggested a poor prognosis of PLAP expressing lung, pancreatic, and colorectal cancers (data available from https://www.proteinatlas.org/ENSG00000163283-ALPP/pathology [26]) would be consistent with a functional role of PLAP in cancer cells. The significant association of PLAP positivity with biologically relevant molecular features such as p16 alterations, GATA3 and p63 expression represents another argument for a non-random development and a potential functional role of PLAP in positive cancer cells.

While our data show that PLAP IHC is not suitable as a prognostic marker in urothelial carcinoma, PLAP positivity in these tumors has important diagnostic and potential therapeutic implications. Given the established role of PLAP IHC as a marker for testicular cancer, the strong PLAP positivity in a subset of urothelial carcinomas deserves attention by pathologists as a potential diagnostic pitfall. The membranous location of PLAP protein in PLAP positive cancers in combination with the complete absence of significant PLAP expression in vital organs of (non-pregnant) humans, makes PLAP a potentially useful therapeutic target. Studies using CAR-T cells or bispecific antibodies targeting the PLAP cell surface protein have been successfully used in preclinical colon and cervical cancer models [9, 27, 28]. If these treatments should prove efficient in clinical patients, urothelial carcinomas would be among the tumors that could best benefit from such new treatments.

In summary, these data show that PLAP is expressed in a significant fraction of pT2-4 urothelial carcinomas, unrelated to cancer aggressiveness but associated with specific molecular features. Once anti-PLAP cancer drugs should become effective, urothelial carcinoma is a candidate tumor entity for clinical evaluation.

## Data Availability

No datasets were generated or analysed during the current study.

## Abbreviations

IHC: Immunohistochemistry
ISUP: International Society of Urological Pathology
IWGAV: International working group for antibody validation
TCGA: The Cancer Genome Atlas
TMA: Tissue microarrays
PLAP: Placental alkaline phosphatase
ALPP: Alkaline phosphatase, placental type
